# Chirality-selective second-harmonic response with Floquet engineering

**DOI:** 10.1126/sciadv.aed6149

**Published:** 2026-10-30

**Authors:** Yi Zhang, Haijun Cao, Youqiang Huang, Luojun Du, Nianze Shang, Xueyin Bai, Xuetao Gan, Dong Mao, Peng Li, Jianlin Zhao, F. Javier García de Abajo, Sheng Meng, Zhipei Sun

**Affiliations:** ^1^ Key Laboratory of Light Field Manipulation and Information Acquisition, Ministry of Industry and Information Technology, and Shaanxi Key Laboratory of Optical Information Technology, School of Physical Science and Technology, Northwestern Polytechnical University, Xi’an 710129, China.; ^2^ Department of Electronics and Nanoengineering, Aalto University, FI-00076 Aalto, Finland.; ^3^ Beijing National Laboratory for Condensed Matter Physics; Key Laboratory for Nanoscale Physics and Devices, Institute of Physics, Chinese Academy of Sciences, Beijing 100190, China.; ^4^ School of Physical Sciences, University of Chinese Academy of Sciences, Beijing 100190, China.; ^5^ ICFO–Institut de Ciencies Fotoniques, The Barcelona Institute of Science and Technology, 08860 Castelldefels (Barcelona), Spain.; ^6^ ICREA–Institució Catalana de Recerca i Estudis Avançats, 08010 Barcelona, Spain.

## Abstract

Symmetry breaking profoundly influences the macroscopic physical properties of materials. For instance, inversion symmetry breaking in monolayer transition metal dichalcogenides enables the emergence of chiral selection rules, featuring distinct optical chirality-selective electronic properties. Here, we demonstrate ultrafast, coherent manipulation of chirality-selective nonlinear optics in monolayer molybdenum disulfide (MoS_2_) through time-reversal symmetry breaking via Floquet engineering. By using sub-bandgap, circularly polarized ultrafast optical pulses, we introduce nondegenerate, chirality-selective optical Stark and Bloch-Siegert shifts. This approach effectively lifts the energy degeneracy, leading to pronounced chirality-selective second-harmonic generation. Furthermore, we reveal that the Floquet-engineered chirality-selective states can be finely tuned using optical parameters such as intensity and chirality. Our results show a method for manipulating the energy degree of freedom, paving the way for ultrafast nonlinear photonics.

## INTRODUCTION

The study of two-dimensional transition metal dichalcogenides (TMDs) has revealed that inversion symmetry breaking dictates the distinct electronic behaviors coupled with chiral light. The engineering and control of time-reversal symmetry can further lift the energy degeneracy for applications in valleytronics and advanced optoelectronics (*1*). In addition to applying an external magnetic field, circularly polarized light (*2*–*5*) or topological light fields (*6*–*8*) can transiently induce the time-reversal and space inversion symmetry breaking, leading to the lifting of energy degeneracy even in multilayer TMDs.

Floquet engineering, a practical technique for achieving coherent light-matter interaction (*9*), has played a key role in atomic physics (*10*, *11*) and quantum optics (*12*–*14*). In chiral optics, Floquet engineering is harnessed to manipulate electrical properties (e.g., spin and pseudospin) in atomically thin quantum materials, including WSe_2_ (*4*), WS_2_ (*2*, *3*), and CsPbI_3_ perovskite (*15*). Furthermore, the Floquet engineering of excitons in monolayer WS_2_ reveals the real-space exciton dynamics in the strong-field regime (*16*). These manipulations are often observed through circular polarization–resolved photoluminescence or absorption spectroscopy in linear optics. Recent advancements highlight strong nonlinear light-matter interactions in layered magnetic insulators like MnPS_3_, where the establishment of Floquet states is evidenced by analytical expressions derived from modified single-ion states, markedly influencing the second-harmonic generation (SHG) process (*17*). Furthermore, the formation and decay dynamics of Floquet states in monolayer semiconductors (*18*) and colloidal nanoplatelets (*19*) have been investigated at room temperature, featuring a few hundred femtoseconds dephasing time. Despite these advancements, the goal of achieving ultrafast coherent control of chiral SHG in layered TMDs—instead of carrier-induced incoherent modulation (*20*, *21*)—has not been realized.

Here, we demonstrate ultrafast coherent manipulation of chirality-selective SHG in monolayer semiconductor MoS_2_ with Floquet engineering. It results in the coherent modulation of optical nonlinearity, an ultrafast parametric process (*17*, *22*) inherently resistant to the photoexcited carriers. Using sub-bandgap circularly polarized pump pulses, we demonstrate the time-reversal symmetry breaking and obtain nondegenerate energy shifts through the optical Stark and Bloch-Siegert effects. We observe distinct ultrafast transient SHG changes of a probe beam, validating the theoretical prediction of Floquet engineering. The demonstrated SHG response for the detection of Floquet-engineered chirality-selective states—manifested through the optical Stark and Bloch-Siegert shifts—contrasts with previous studies that relied on linear optical techniques such as transient reflection or absorption spectroscopy (*2*–*4*), and it opens a different pathway for the coherent probing and manipulation of energy degeneracy. This method enables coherent manipulation of energy degeneracy within ultrafast speed (less than hundreds of femtoseconds) and holds substantial potential for coherent nonlinear optoelectronic applications in quantum technologies.

## RESULTS

### Floquet-engineered chirality-selective optical nonlinearity

For monolayer semiconducting TMDs, it is justified to model the fundamental excitonic transitions (denoted as ∣a⟩ and ∣b⟩) as a two-level system, separated by an energy gap *E*_0_ (Fig. 1, A and B) at a certain crystal momentum **k** under the assumption of coherent light-matter interaction. When a TMD is driven by the oscillating electric field of a sub-bandgap light with frequency ω (i.e., ℏω < *E*_0_, ℏ is the reduced Planck’s constant), it creates evenly spaced photon-dressed states, known as Floquet states (*9*). These states are separated by ∼ℏω. For instance, under the first-order approximation, when the TMD is driven by a sub-bandgap pump beam with a left-handed (σ^−^) circular polarization, two distinct types of Floquet states emerge in the driven system (*2*): (i) appearing between the two original energy states with a corotating driving field, manifests as the optical Stark effect (Fig. 1A, left), and (ii) emerging outside the original energy range with a counterrotating driving field, is recognized as the Bloch-Siegert effect (Fig. 1B, left). Note that similar effects (i.e., optical Stark effect with a counterrotating driving field and Bloch-Siegert effect with a corotating driving field) occur when the pump light is a sub-bandgap right-handed (σ^+^) circular polarization (fig. S1).

**Fig. 1.**
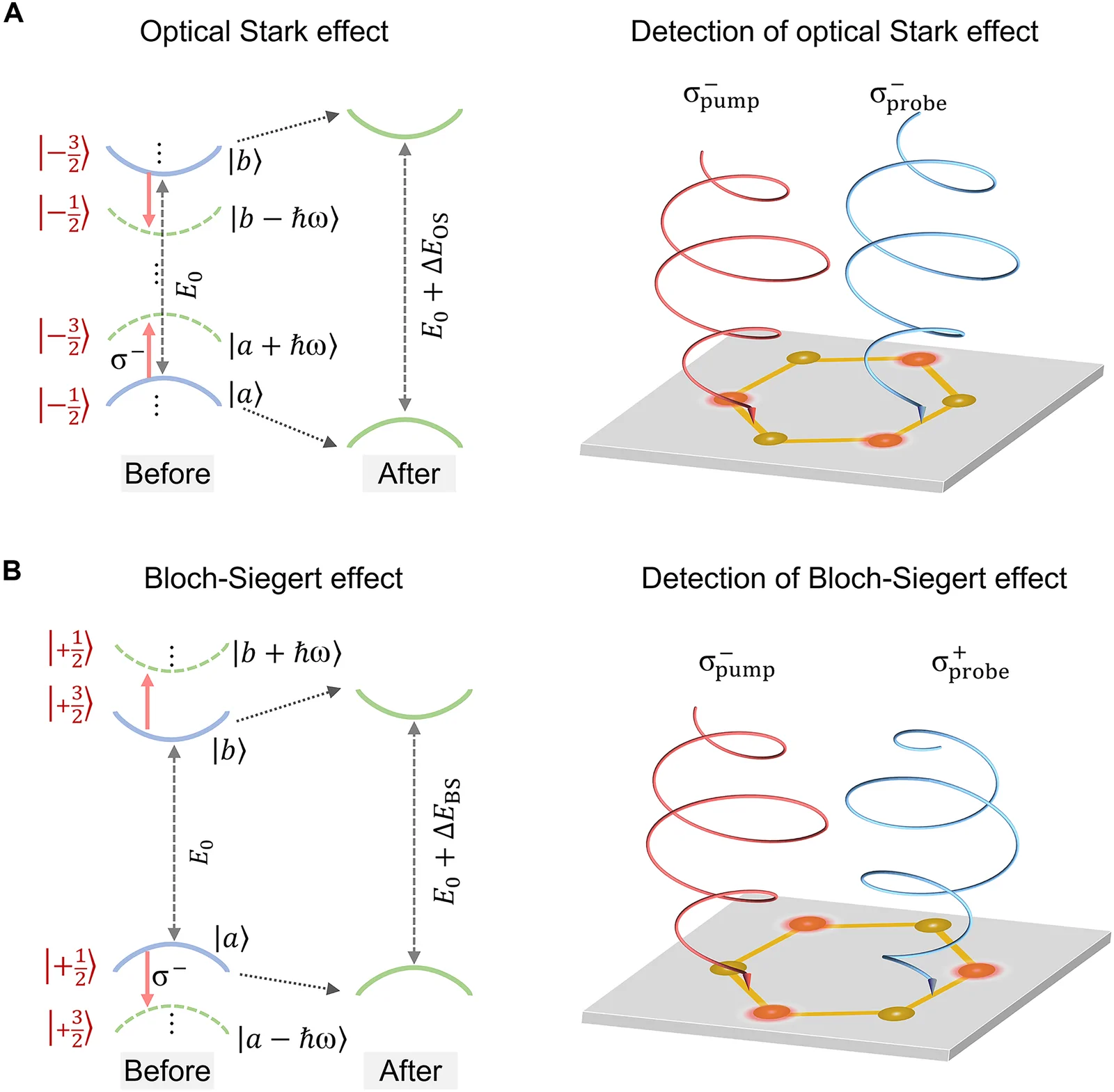
Floquet-engineered time-reversal symmetry breaking and chirality-selective probe schemes. (**A**) Energy diagram illustrating the optical Stark effect–induced energy shift (Δ*E*_OS_) (left). (**B**) Energy diagram illustrating the Bloch-Siegert effect–induced energy shifts (Δ*E*_BS_) (left), when a TMD is driven by a sub-bandgap pump beam with σ^−^ circular polarization (pink arrows). Right: Chirality-selective pump-probe schemes for detecting Floquet states. Note that the lifted energy degeneracy is highlighted by rendering atoms in red. The numbers in red associated with energy states in (A) and (B) are magnetic quantum numbers (∣m⟩). Ellipses among energy levels indicate omitted higher-order Floquet states.

In the following discussion, we will further explore the case in which the TMD, specifically monolayer MoS_2_, is driven by a sub-bandgap σ^−^ circularly polarized pump beam. The magnetic quantum number of the highest occupied (lowest unoccupied) state ∣a⟩ (∣b⟩) in the valence (conduction) band is *m* = −1/2 (−3/2) and *m* = +1/2 (+3/2) at reciprocal **k** states, respectively (*2*) (Fig. 1, left). Under strong electric field driving, a series of Floquet states, denoted as ∣a±ℏω⟩ and ∣b±ℏω⟩, are generated, in which higher-order terms are omitted due to trivial contributions. The magnetic quantum numbers of the generated Floquet states follow a change Δ*m* = 1 (−1) for the energy descending (ascending) cases, corresponding to the strong electric field driving and stimulated emission of light (*3*). The resulting Floquet states will repel original equilibrium states considering the proximity in energy and matching of magnetic quantum number (i.e., angular momentum conservation), by analogy with orbital hybridization in Coulomb interactions. The repulsion between the created Floquet state in the valence band with the same magnetic quantum number as that of the conduction band results in an enlarged energy gap *E*_0_ + Δ*E*_OS_ (*E*_0_ + Δ*E*_BS_) due to the corotating (counterrotating) driving field, where Δ*E*_OS_ ∝ *I*_ω_/(*E*_0_ − ℏω) [Δ*E*_BS_ ∝ *I*_ω_/(*E*_0_ + ℏω)] represents the optical stark (Bloch-Siegert) shift as shown in the left panel of Fig. 1, and *I*_ω_ is the incident light intensity.

The interactions between the Floquet states of valence and conduction bands induce the time-reversal symmetry breaking between reciprocal **k**-states (*23*). It allows the coherent detection of energy degeneracy broken using a probe beam with either corotating or counterrotating circular polarizations. Specifically, when a TMD is driven by a sub-bandgap pump beam with σ^−^ circular polarization (pink input), a probe beam with σ^−^ circular polarization (blue input) detects the Floquet states induced by the optical Stark effect (Fig. 1A, right). Conversely, a probe beam with σ^+^ circular polarization (blue input) detects the Floquet states induced by the Bloch-Siegert effect (Fig. 1B, right). To detect transient Floquet states, we use second-order optical nonlinearity $\chi^{\left(2\right)}$, which has been previously used to detect the energy level shifting (*17*, *24*). To account for SHG variation induced by Floquet states, we use a microscopic expression of $\chi^{\left(2\right)}$ using a time-dependent perturbation theory (*25*)

$$\chi_{\mathrm{ijk}}^{\left(2\right)}\propto \langle g|{\hat{V}}_{i}^{2\omega }\frac{|f\rangle \langle f|}{E_{f}-2\omega \hbar -i\Gamma_{f}}{\hat{V}}_{j}^{\omega }\sum_{m}\frac{|m\rangle \langle m|}{E_{m}-\omega \hbar -i\Gamma_{m}}{\hat{V}}_{k}^{\omega }|g\rangle$$ (1)

where ∣g⟩, ∣m⟩, and ∣f⟩ represent the ground, intermediate, and final states in the SHG process, respectively, with *E_g_*, *E_m_*, and *E_f_* denoting their respective energies. $\Gamma_{f}$ and $\Gamma_{m}$ are the damping rates for the final and intermediate states with $\Gamma_{f}\ll \Gamma_{m}$ as the excitonic final state lives remarkably longer than a virtual intermediate state. The indices *i*, *j*, and *k* stand for the σ^−^, σ^+^ circularly polarized, and *z*-polarized pump beam, respectively, where *z* is normal to monolayer MoS_2_ as indicated in Fig. 2A, ${\hat{V}}_{i}^{\omega }$ denotes the exciton-photon interaction terms (detailed expressions in section S2). The nondegenerate Floquet-engineered states, caused by differences in the optical Stark and Bloch-Siegert shifts, lead to variations in the reduced $\chi^{\left(2\right)}$. This results in chirality-selective SHG modulation, which is modeled in detail by the Floquet theory in Materials and Methods.

**Fig. 2.**
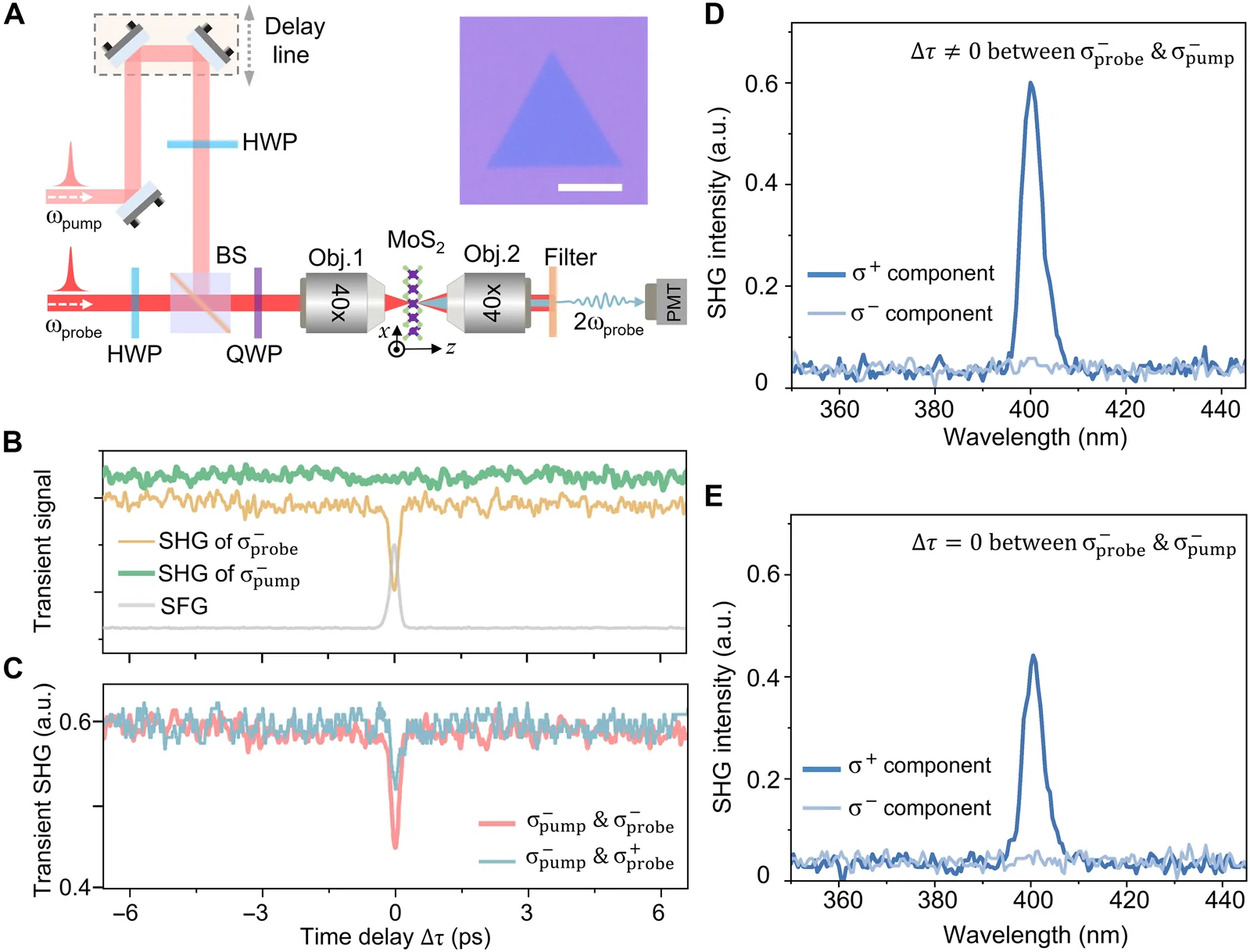
Observation of Floquet-engineered chirality-selective transient SHG in monolayer MoS_2_. (**A**) Schematic illustration of the experiment. We pump monolayer MoS_2_ with a near-infrared pump beam and measure Floquet state–induced changes in the SHG of a probe beam. HWP, half-wave plate; QWP, quarter-wave plate; BS, beam splitter; PMT, photomultiplier tube. The inset is a microscope image of monolayer MoS_2_. Scale bar, 20 μm. (**B**) Transient SHG of the pump (green line) and the probe (orange line) beams. Both beams are σ^−^ circularly polarized, and their SFG intensity (gray line) is recorded as a reference signal to determine the zero-time delay position between them. Note that the SHG and SFG signals are shifted vertically for a clear view. (**C**) Measured chirality-selective transient SHG of a σ^−^ or σ^+^ circularly polarized probe beam in the presence of a σ^−^ circularly polarized pump beam. a.u., arbitrary units. (**D** and **E**) Circular polarization–resolved components of the SHG spectra of the σ^−^ probe beam when it is nonsynchronized (D) or synchronized (E) with the same circularly polarized pump beam. The intensity of the pump (at a wavelength of ∼1000 nm) and probe beam (at a wavelength of ∼800 nm) in all cases are fixed at ∼15 GW/cm^2^.

### Observation of Floquet-engineered chirality-selective transient SHG

To experimentally detect the prediction of nondegenerate states induced by Floquet engineering in monolayer MoS_2_ with nonlinear optics, we use a typical pump-probe experiment setup to observe the time-resolved SHG of a probe beam (Fig. 2A; described in Materials and Methods). All experiments are implemented at room temperature. Notably, the used pump wavelength is selected far below the bandgap of monolayer MoS_2_, ∼1.85 eV (*26*), facilitating the creation of Floquet states (*17*, *27*). Moreover, the pump light is selected as a σ^−^ circular polarization, whereas the probe light can be either a σ^−^ or σ^+^ circular polarization to detect Floquet-engineered energy bands (Fig. 1). We use the SHG of the probe beam to investigate chirality-selective optical nonlinearity due to the Floquet-engineered states (discussed above). The synchronization between the pump and probe beams is confirmed by the sum-frequency generation (SFG), showing an intensity peak at the zero-time delay Δτ = 0 (gray line in Fig. 2B). Note that the SHG of the pump beam remains constant regardless of the time delay (green line in Fig. 2B), indicating an absence of modulation. This constancy suggests that the SHG of the pump beam is not subject to modulation through photon-excited carriers (*20*, *21*). In our case, the SHG of the probe beam is notably modulated by the presence of the pump beam (orange line in Fig. 2B). This modulation is an ultrafast process, coinciding with the pulse duration, ∼183 fs of full width at half maximum (FWHM), of the SFG signal. The probe beam has a negligible influence on the pump beam’s SHG signal (green line in Fig. 2B). This is because its shorter wavelength leads to a negligible Floquet energy shift, a phenomenon that has also been observed in monolayer WS_2_ using transient absorption spectroscopy (*3*).

Figure 2C shows the transient SHG at different probe chiralities. The SHG change Δ*I*/*I*_0_ is ∼24% when the probe and pump beams have the same optical chirality, whereas Δ*I*/*I*_0_ is ∼13% when the probe and pump beams have different optical chiralities. Here, *I*_0_ and *I*_pump_ are the SHG intensities of the probe beam without and with the pump beam, and Δ*I* = *I*_0_ − *I*_pump_, where *I*_pump_ is a transient suppression of the SHG response (i.e., *I*_pump_ < *I*_0_). Consequently, the modulation depth ∆*I*/*I*_0_ remains positive. The difference (up to ∼2 times) in the SHG changes at different probe chiralities is primarily because the optical Stark shift Δ*E*_OS_ is larger than the Bloch-Siegert shift Δ*E*_BS_ (detailed discussion later). This effectively confirms the lifting of energy degeneracy with different driving fields. Moreover, the decay and recovery of the transient SHG change are symmetric. This symmetry signifies a coherent energy shift and excludes any obvious contribution from carriers’ scattering, which typically exhibits asymmetric dynamics (*28*, *29*).

We also characterize the polarization components (i.e., chirality) of the SHG of the σ^−^ probe beam, which always shows opposite chirality to the fundamental probe beam regardless of the presence of the σ^−^ pump beam (Fig. 2, D and E). This chiral selection rule of the SHG process is dictated by the conservation of angular momentum (*30*). This further indicates that the observed SHG modulation is a coherent process driven by the energy level shifting induced by Floquet-engineered states on an ultrafast timescale. The transient SHG duration (∼183 fs of FWHM) matches the pump pulse width, precluding the resolution of Floquet states on a time scale shorter than 100 fs (*18*, *31*). This implies that the observed temporal response is essentially pulse width limited. However, the symmetry of the time-resolved signal confirms the absence of long-lived incoherent hot-carrier effects, supporting the coherent and virtual nature of Floquet dressed states. In contrast, conventional approaches to modulating optical nonlinearity, which rely on photocarriers, typically operate on a picosecond timescale (or even longer) due to the relatively slow electron-hole relaxation processes (*28*, *29*). Note that the recently reported coherent modulations of SHG (*32*) and third-harmonic generation (*33*) were achieved by the coherent superposition of two distinctly polarized beams at the same wavelength instead of creating Floquet states. These methods, therefore, do not allow for the tuning of quantum effects of low-dimensional nanomaterials, unlike our Floquet-engineered approach.

### All-optical engineering of chirality-selective optical nonlinearity

The Floquet-engineered energy level shifting (i.e., Δ*E*_OS_ and Δ*E*_BS_) depends on the intensity (i.e., *I*_ω_) and chirality of the pump beam. Therefore, we anticipate that the Floquet-engineered states introduced transient SHG change Δ*I*/*I*_0_ could also be modulated by both the intensity and chirality of the pump beam. To study these relationships, we first fix the intensity of the probe beam (∼15 GW/cm^2^) and change the intensity of the pump beam with ℏω_pump_ ∼ 1.24 eV. Figure 3A shows the simulated optical Stark shift when the pump beam is σ^−^ circularly polarized, which shows a linear dependence on the intensity of the pump beam as demonstrated in previous works (*2*, *3*). We simulate the energy shift effect on SHG via first-principles calculations based on density functional theory (DFT) using the projector augmented wave method (*34*) implemented in the Vienna ab initio simulation package (VASP) (*35*). The calculation details are given in Materials and Methods. The measured transient SHG change Δ*I*/*I*_0_ (red squares) under the same optical chirality of the probe and pump beams is shown in Fig. 3B, which shows a monotonically but nonlinear growth as a function of the pump intensity. A maximum modulation depth of ∼35% is achieved at the pump power of ∼21 GW/cm^2^. To better understand the origin of the transient SHG change, the calculation results (green curve in Fig. 3B) considering the optical Stark effect are given, which exhibit good agreement with experimental results. The thermal influence on the Floquet states is negligible in our case for two main reasons. First, the off-resonant pump photon energy is far below the bandgap of monolayer MoS_2_, resulting in minimal energy absorption, as demonstrated in linear optical studies (*3*). Second, the heating effect induced by the femtosecond pump pulse is minimal due to the low pump power used in our experiment, ensuring that the deposited energy is insufficient to cause any noticeable changes in the atomic structure. In addition, the pump-induced nonlinear refractive index change (i.e., Kerr effect) is negligible in monolayer MoS_2_ (*36*). Consequently, its contribution to the SHG intensity change is negligible compared to that induced by Floquet engineering.

**Fig. 3.**
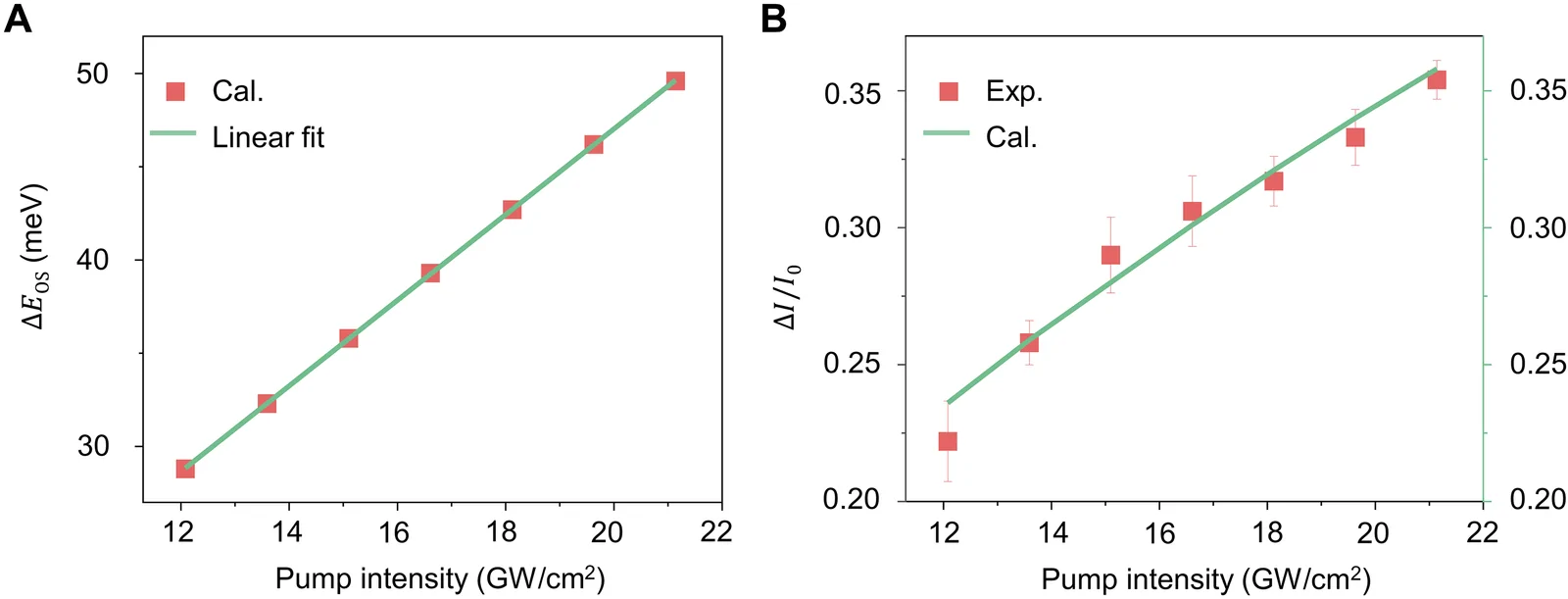
Optical Stark effect–induced transient SHG. (**A**) Calculated optical Stark shift as a function of pump intensity. (**B**) Transient SHG intensity change Δ*I*/*I*_0_ of a σ^−^ circularly polarized probe beam at a wavelength of ∼800 nm versus the intensity of a σ^−^ circularly polarized pump with ℏω_pump_ ∼ 1.24 eV (a wavelength of ∼1000 nm). Error bars represent the SEM value calculated from three separate measurements.

Next, we switch the optical chirality of the pump beam to study the Bloch-Siegert effect (Fig. 4). The calculated Bloch-Siegert effect–induced energy shift Δ*E*_BS_ is also shown in Fig. 4A, agreeing well with the previously reported values measured by the reflection (*4*) or photoluminescence (*2*) measurements. In Fig. 4B, the measured transient SHG change also agrees with the calculation results, featuring a monotonically but nonlinear growth. The changes in SHG intensity (Figs. 3B and 4B) represent a nonlinear transduction of the linear energy shifts depicted in Figs. 3A and 4A, in accordance with Eq. 1. Note that the calculation parameters are compromised to match the optical Stark effect–induced SHG change, which inevitably leads to small deviations from the Bloch-Siegert effect–induced SHG change mainly due to the uncertainty of resonance excitonic states (*37*). The calculated transient SHG changes caused by different excitonic states are shown in section S3. We observe a transient SHG change Δ*I*/*I*_0_ of ∼22% at the pump intensity of ∼25 GW/cm^2^ (Fig. 4B). This is smaller than that induced by the optical Stark effect due to the smaller Bloch-Siegert shift Δ*E*_BS_ (Fig. 4A). Nevertheless, it provides an extra knob (i.e., chirality) to coherently tune the optical nonlinearity compared to previously demonstrated noncoherent methods. Moreover, the transient SHG change can provide insights into the Floquet states shift behaviors. Such capability is particularly promising for applications in nonlinear chiral quantum optics (*38*).

**Fig. 4.**
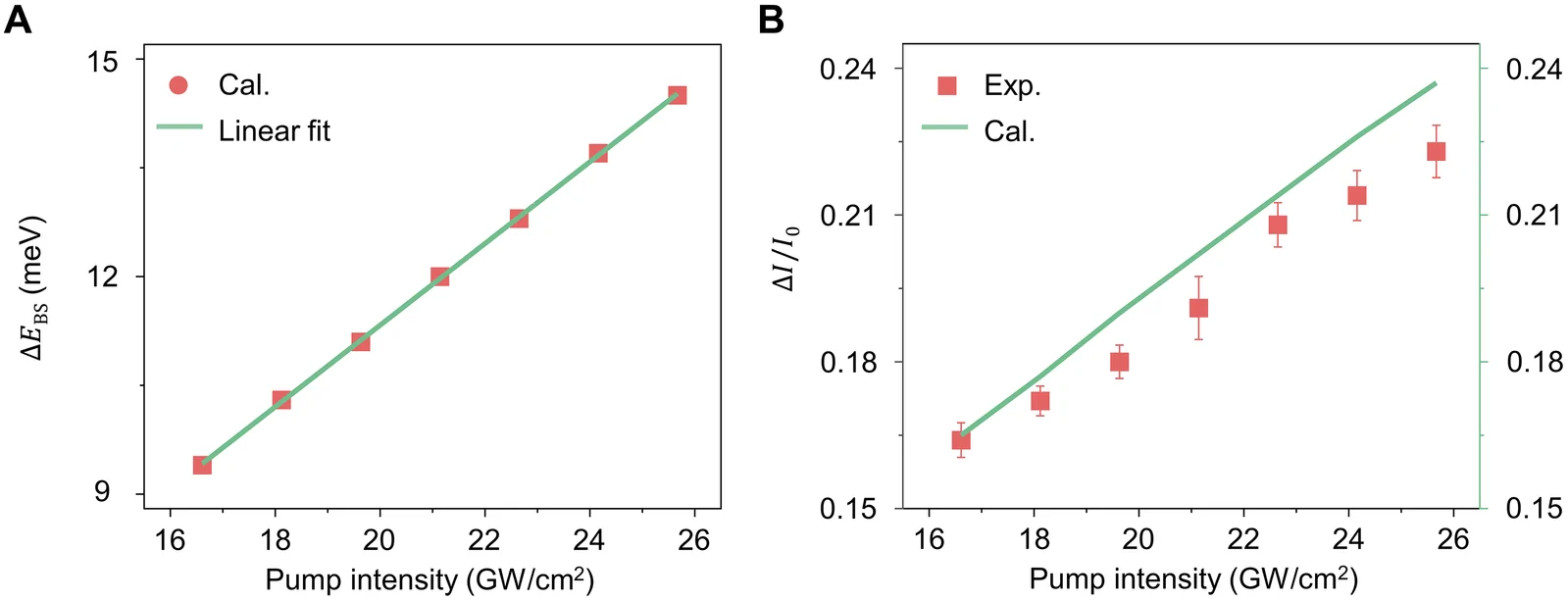
Bloch-Siegert effect–induced transient SHG. (**A**) Calculated Bloch-Siegert shift as a function of pump intensity. (**B**) Transient SHG intensity change Δ*I*/*I*_0_ of a σ^−^ circularly polarized probe beam at a wavelength of ∼800 nm versus the intensity of a σ^+^ circularly polarized pump with ℏω_pump_ ∼ 1.24 eV (a wavelength of ∼1000 nm). Error bars represent the SEM value calculated from three separate measurements.

## DISCUSSION

In summary, we demonstrate the chirality-selective SHG in monolayer semiconductor MoS_2_ with Floquet engineering. Using a sub-bandgap circularly polarized pump light, the induced unequal optical Stark and Bloch-Siegert energy shifts lead to the time-reversal symmetry breaking in MoS_2_. The different energy level shifts are characterized by a transient SHG change of the probe beam with different circular polarization. The modulation depth of SHG increases monotonically with the pump power and is explained by calculating the microscopic second-order nonlinear susceptibility considering the contributions from the energy level shifting. The ab initio calculations further consolidate the observed results. The observed chirality-selective SHG also provides an ultrafast method to coherently manipulate the energy degeneracy in TMDs with multiple optical parameters (e.g., pump intensity and chirality). Note that the contrast transient SHG outputs by changing the chirality combination of pump and probe beams are also ideal for constructing chirality logic gates (*39*). Moreover, the transient SHG change comes from the energy level shift–induced modification of nonlinear susceptibility instead of absorption (as demonstrated with a different pump wavelength in fig. S5) or defects (as demonstrated with the mechanically exfoliated MoS_2_ flake in fig. S6), which is inherently resistant to photoexcited excitons and carriers’ scattering and thus holds the potential to detect and manipulate the Floquet chiral edge states (*3*).

## MATERIALS AND METHODS

### Sample preparation

Monolayer MoS_2_ flakes are grown on a 500-μm-thick quartz substrate (Siegert wafer) using the chemical vapor deposition (CVD) method. A Na_2_MoO_4_ aqueous solution (5 mg/ml) is spin coated on top of the substrate and is heated at 800°C, and 10 mg of sulfur is heated at 170°C for 5 min in high-purity argon. The optical microscope image (inset of Fig. 2A) and Raman and photoluminescence spectroscopies (fig. S7) evidence high-quality monolayer MoS_2_ flakes (*40*, *41*).

### Transient SHG experiment setup

The pump and probe beams for the experiment (see Fig. 2A) are generated by a laser source with a repetition rate of 2 kHz from an amplified Ti:sapphire femtosecond laser system (Spectra-Physics Solstice Ace). We use the half-wave plate (HWP) to control the polarization orientation of the pump and probe beams. After passing through a delay line system, the pump beam is collinearly combined with the probe beam by a nonpolarizing beam splitter. The pump and probe beams are synchronized temporally by moving the delay line. We use a quarter-wave plate (QWP) to convert the linearly polarized pump and probe beams into circularly polarized beams. After passing through a 40x objective lens [numerical aperture (NA) = 0.75; Nikon], the circularly polarized pump and probe beams are focused onto the MoS_2_ monolayer. The spot size of the pump and probe beam in our measurement is ∼2 μm. The SHG signal of the probe beam is collected by another 40x objective lens (NA = 0.5; Nikon) and relayed to a spectrometer (Andor) equipped with a photomultiplier tube (Hamamatsu), which is connected to a lock-in amplifier (SR830, Stanford Research Systems). This frequency selection technique ensures that the detected SHG signal is immune to external interference from other frequency components and noises, thereby maintaining a high signal-to-noise ratio. Two 700-nm short-pass filters (Edmund) after the second objective lens are used to block the pump and probe beams.

### First-principles calculations

The first-principles calculations based on DFT using the projector augmented wave method (*34*) are implemented in the VASP (*42*) in our calculations to obtain the Hamiltonian of monolayer MoS_2_. The generalized gradient approximation (GGA) with the Perdew-Burke-Ernzerhof (PBE) exchange-correlation functional (*43*) is used for lattice parameter optimization. To calculate the electronic properties, the Heyd-Scuseria-Ernzerhof hybrid function (*44*) is used. The detailed computational parameters for calculating intrinsic Hamiltonian are as follows. The kinetic energy cutoff is set to 500 eV for the plane-wave basis, and a Г-centered 12×12×1 *k*-point mesh is used in the first Brillouin zone. The convergence criteria for the ionic relaxation loop and electronic self-consistent loop are set to be 0.01 eV/Å and 10^−6^ eV, respectively.

### Floquet Hamiltonian

To obtain the Floquet Hamiltonian, we first construct the Hamiltonian H^W^(***k***) in equilibrium on a Wannier basis by using the Wannier90 interface (*35*, *45*, *46*). The time-periodic laser field (i.e., the pump light field) is incorporated into the Hamiltonian by the Peierls substitution (*47*)

$$H^{W}\left(k,t\right)=H^{W}\left[k+\frac{e}{\hbar }A\;\left(t\right)\right]$$ (2)

where $A\left(t\right)=A_{0}\left(\text{sinω}t,\pm \text{cosω}t\right)$ represents the vector potential of the circularly polarized pump field with an amplitude *A*_0_ and a frequency ω. The field strength is selected according to the experimental conditions. The electronic properties in the light-driven solids are governed by the Schrödinger equation (*48*, *49*)

$$i\hbar \frac{\partial }{\partial t}|\psi \left(k,t\right)\rangle =H^{W}\left(k,t\right)|\psi \left(k,t\right)\rangle$$ (3)

Because the pump laser is periodic, according to the Floquet theorem (*45*–*47*), the electronic wave function, solution of Eq. 3, can be expressed as

$$|\psi \left(k,t\right)\rangle =e^{-i\varepsilon \left(k\right)t/\hbar }|\Phi \left(k,t\right)\rangle$$ (4)

Here, ε(k) is the Floquet quasienergy and ∣Φ(k,t)⟩ is the Floquet mode with the period $T=\frac{2\pi }{\omega }$ and ∣Φ(k,t)⟩=∣Φ(k,t+T)⟩. We expand the ∣Φ(k,t)⟩ as discrete Fourier series

$$\left|\Phi \left(k,t\right)\rangle =\sum_{n=-\infty }^{\infty }e^{-\text{in}\omega t}\right|u^{n}\left(k\right)\rangle$$ (5)

where n is an integer and can be taken as the number of photons. Substituting Eqs. 4 and 5 into Eq. 3, we can transform the Schrödinger equation into a time-independent equation

$$\sum_{n}H_{m,n}\left(k\right)|u^{n}\left(k\right)\rangle =\left[\varepsilon \left(k\right)+m\hbar \omega \right]|u^{m}\left(k\right)\rangle$$ (6)

The matrix element $H_{m,n}\left(k\right)$ can be obtained by averaging the time-dependent Hamiltonian $H^{W}\left(k,t\right)$ over one period of the oscillating field

$$H_{m,n}\left(k\right)=\frac{1}{T}\int_{0}^{T}H^{W}\left(k,t\right)e^{i\left(m-n\right)\omega t}\mathrm{dt}$$ (7)

Thus, Eq. 6 can be rewritten as

$$\sum_{n}{\left(H_{F}^{W}\right)}_{m,n}\left(k\right)|u^{n}\left(k\right)\rangle =\varepsilon \left(k\right)|u^{m}\left(k\right)\rangle$$ (8)

Therefore, we can get the Floquet Hamiltonian that explicitly incorporates the amplitude and frequency of the applied pump light field

$$H_{F}^{W}\left(k\right)=\left[\begin{array}{ccccc} \ddots & \vdots & \vdots & \vdots & ⋰\\ \cdots & H_{-1,-1}\left(k\right)+\hbar \omega & H_{-1,0}\left(k\right) & H_{-1,1}\left(k\right) & \cdots \\ \cdots & H_{0,-1}\left(k\right) & H_{0,0}\left(k\right) & H_{0,1}\left(k\right) & \cdots \\ \cdots & H_{1,-1}\left(k\right) & H_{1,0}\left(k\right) & H_{1,1}\left(k\right)-\hbar \omega & \cdots \\ ⋰ & \vdots & \vdots & \vdots & \ddots \end{array}\right]$$ (9)

In our calculations, $H_{F}^{W}\left(k\right)$ is truncated to the fifth order (*n* = 0, ±1, ±2). By numerically diagonalizing the Floquet Hamiltonian, we obtained the field-dressed band structure and, consequently, the corresponding quasienergy shifts.

### Floquet state–induced SHG

To describe the chirality-selective SHG response, we use a sum-over-states model for the second-order nonlinear susceptibility $\chi^{\left(2\right)}$ (i.e., Eq. 1) where the transition energies *E_m_*/*E_f_* are modified by the strong-field driving as $\Delta E\propto {\alpha I}_{\omega }$. As shown in Figs. 3A and 4A, the underlying energy level shifts scale linearly with the pump intensity in this off-resonant regime. According to Eq. 1, the second-order nonlinear susceptibility is modified as the excitonic state is shifted by ΔE. When the shift is incorporated into the resonance denominators of $\chi^{\left(2\right)}$, the resulting SHG intensity change (Δ*I*/*I*_0_) can be written as (see section S3 for detailed derivation)

$$\frac{\Delta I}{I_{0}}=1-\frac{\Delta_{0}^{2}+\Gamma_{f}^{2}}{{\left(\Delta_{0}+\alpha I_{\omega }\right)}^{2}+\Gamma_{f}^{2}}$$ (10)

where $\Delta_{0}=E_{f}-2\hbar \omega$ represents the effective detuning between the probe photon energy and the relevant excitonic resonance. The theoretical results, represented by the solid green lines in Figs. 3B and 4B, were obtained according to Eq. 10. They both exhibit nonlinear dependence on the pump intensity as seen in the curvature of the green lines in Figs. 3B and 4B. This nonlinearity arises because the SHG depends on the square of the susceptibility and is highly sensitive to the detuning relative to the band-nesting region. The transient SHG changes with different excitonic states are provided in section S3.

## Supplementary Materials

### This PDF file includes:

Sections S1 to S6 Figs. S1 to S7
