# Supplementary material for: Chirality-selective second-harmonic response with Floquet engineering
Source: Sci Adv. 2026 Oct 30;12(44):eaed6149. doi: 10.1126/sciadv.aed6149 (PMC13721968; doi:10.1126/sciadv.aed6149)
Supplement: Supplementary file 1 [file sciadv.aed6149_sm.pdf]

Science Advances
AAAS

Supplementary Materials for

Chirality-selective second-harmonic response with Floquet engineering

Yi Zhang *et al.*

Corresponding author: Yi Zhang, yi.zhang@nwpu.edu.cn; Sheng Meng, smeng@iphy.ac.cn;
Zhipei Sun, zhipei.sun@aalto.fi

Sci. Adv. **12**, eaed6149 (2026)
DOI: 10.1126/sciadv.aed6149

This PDF file includes:

Sections S1 to S6
Figs. S1 to S7

S1 Floquet-engineered time-reversal symmetry breaking

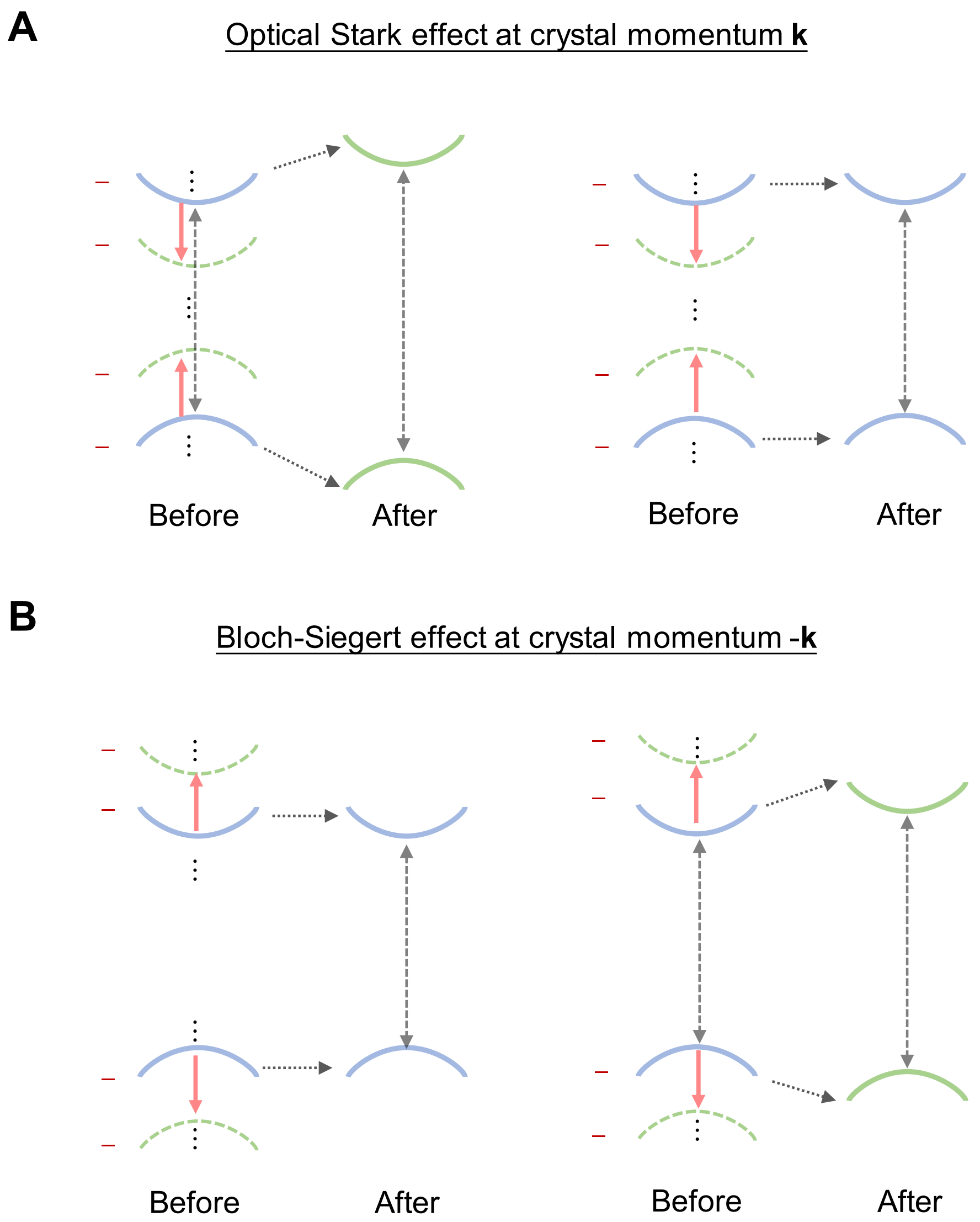

Fig. S1 Comparison of Optical Stark shift and Bloch-Siegert shift in reciprocal k-states. (A) Energy diagram before and after a sub-bandgap pump beam with σ^{-} circular polarization (pink arrows) (co-rotating driving field), the hybridization between Floquet-induced energy states causes an energy shift ΔE_{OS} (left panel) due to the optical Stark effect. **(B)** Energy diagram before and after a sub-bandgap pump beam with σ^{-} circular polarization (pink arrows) (counter-rotating driving field), the hybridization between Floquet-induced energy states causes an energy shift ΔE_{BS} (right panel) due to the Bloch-Siegert effect.

S2 Microscopic calculations of the second-order nonlinear susceptibility

The general form of the second-order nonlinear susceptibility $\chi^{(2)}$ in the vicinity of the exciton resonance energy, based on time-dependent perturbation theory, can be given as

$$\chi_{ijk}^{(2)} \propto \langle g|\hat{V}_i^{2\omega} \frac{|f\rangle\langle f|}{E_f - 2\omega\hbar - \mathrm{i}\Gamma_f} \hat{V}_j^{\omega} \sum_m \frac{|m\rangle\langle m|}{E_m - \omega\hbar - \mathrm{i}\Gamma_m} \hat{V}_k^{\omega}|g\rangle \tag{S1}$$

where $|g\rangle$, $|m\rangle$, and $|f\rangle$ represent the ground, intermediate, and final states in the SHG process, respectively, with E_g, E_m, and E_f denoting their respective energies. Γ_f and Γ_m are the damping rates for the final and intermediate states with $\Gamma_f \gg \Gamma_m$, as the excitonic final state lives significantly longer than a virtual intermediate state. The indices *i*, *j*, and *k* stand for the σ^-, σ^+ circularly polarized, and *z*-polarized pump beams, respectively. For a pump beam perpendicular to the sample plane (*xy*-plane), the exciton-photon interaction term can be expressed as

$$\hat{V}_i^{\omega} = \hat{V}_i^{\mathrm{ED}}(\omega) \quad \hat{V}_i^{\mathrm{EQ}}(\omega) \quad \hat{V}_i^{\mathrm{MD}}(\omega) + h.c. \tag{S2}$$

where "*h.c.*" means the "Hermitian conjugate" operation. Here, $\hat{V}_i^{\mathrm{ED}}(\omega)$, $\hat{V}_i^{\mathrm{EQ}}(\omega)$, and $\hat{V}_i^{\mathrm{MD}}(\omega)$ are the electric dipole, electric quadrupole, and magnetic dipole interactions, respectively:

$$\hat{V}_i^{\mathrm{ED}}(\omega) = -\mathrm{i}\frac{\varepsilon}{2m_0 c} e^{\mathrm{i}\omega t} \mathbf{e}_\perp \cdot \hat{\mathbf{p}}_\perp$$

$$\hat{V}_i^{\mathrm{EQ}}(\omega) = \frac{q_z \varepsilon_0}{2m_0 c} e^{\mathrm{i}\omega t}[(\mathbf{e}_z \cdot \hat{\mathbf{r}})(\mathbf{e}_\perp \cdot \hat{\mathbf{p}}_\perp) + (\mathbf{e}_\perp \cdot \hat{\mathbf{r}}_\perp)(\mathbf{e}_z \cdot \hat{\mathbf{p}}_z)] \tag{S3}$$

$$\hat{V}_i^{\mathrm{MD}}(\omega) = \frac{q_z \varepsilon}{2m_0 c} e^{\mathrm{i}\omega t} \mathbf{e}_z \cdot [\mathbf{e}_\perp \times (\hat{\mathbf{L}} \quad 2\hat{\mathbf{S}})]$$

where $\mathbf{e}_\perp$ and $\mathbf{e}_z$ represents the in-plane and out-of-plane electric field vector of the fundamental wave, $q_z = q = \omega/c$, $\hat{\mathbf{p}}$ is the momentum operator, $\hat{\mathbf{L}}$ is the orbital momentum operator, $\hat{\mathbf{S}}$ is the spin momentum operator. Note that the electric quadrupole is an odd function with respect to the *z*-axis, the resulting interactions with energy states vanish; the orbital part of the magnetic dipole is active, while its spin-dependent part only couples to non-optically active states.

S3 Excitonic state effect on the SHG intensity

As discussed, we attribute the change of SHG intensity to the exciton energy shifts due to the far below band gap of the pump photon energy. We can approximate the second-order susceptibility to $\chi^{(2)} \propto \frac{}{E_f - 2\hbar\omega - i\Gamma_f}$. Under the irradiation of a pump light, the optical Stark or Bloch-Siegert shift is denoted as ΔE. In the off-resonant regime, the shift scales linearly with the pump intensity $\Delta E \propto \alpha I_\omega$, confirmed by our ab initio calculations as shown in Figs. 3A and 4A. When the excitonic state is shifted by ΔE, the susceptibility changes from $\chi^{(2)} = \frac{}{E_f - \ \hbar\omega - \mathrm{i}\Gamma_f}$ to $\chi^{(2)\prime} = \frac{}{(E_f + \Delta E) - \ \hbar\omega - \mathrm{i}\Gamma_f}$. Then, the SHG intensity can be calculated as $I_{2\omega} \propto | \chi^{(2)} |^2$. Therefore, the relative change of the SHG signal can be written as

$$\frac{\Delta I}{I_0} = \frac{I_0 - I_{\mathrm{pump}}}{I_0} = \frac{|\chi^{(2)}|^2 - |\chi^{(2)\prime}|^2}{|\chi^{(2)}|^2} = 1 \quad \frac{\Delta_0^2 + \Gamma_f^2}{(\Delta_0 + \alpha I_\omega)^2 + \Gamma_f^2} \qquad \text{(S4)}$$

where $\Delta_0 = E_f \quad 2\hbar\omega$, representing the effective detuning between the probe photon energy and the relevant excitonic resonance. In out calculations, Δ_0 is set as ~ 0.1 eV, 0.15 eV, 0.2 eV, and the damping rate of the excitonic states is set as Γ_f~3 meV, considering the typical relaxation time of ~100 fs in our calculation. Figure S2 shows the transient SHG change under different excitonic energies and a fixed damping rate. The variation of $\Delta I/I_0$ shares the same trend with the pump beam intensity at different excitonic energies, and the magnitude of $\Delta I/I_0$ decreases when $E_f - 2\hbar\omega$ increases. Note that the optical Stark shift and Bloch-Siegert shift are independent of the excitonic states and the damping rate, which is implied in the top horizontal axis in Fig. S2. We employ the blue and grey curves in Fig. S2A and S2B, respectively, to achieve a better match with the experimental results in Fig. 3B and 4B.

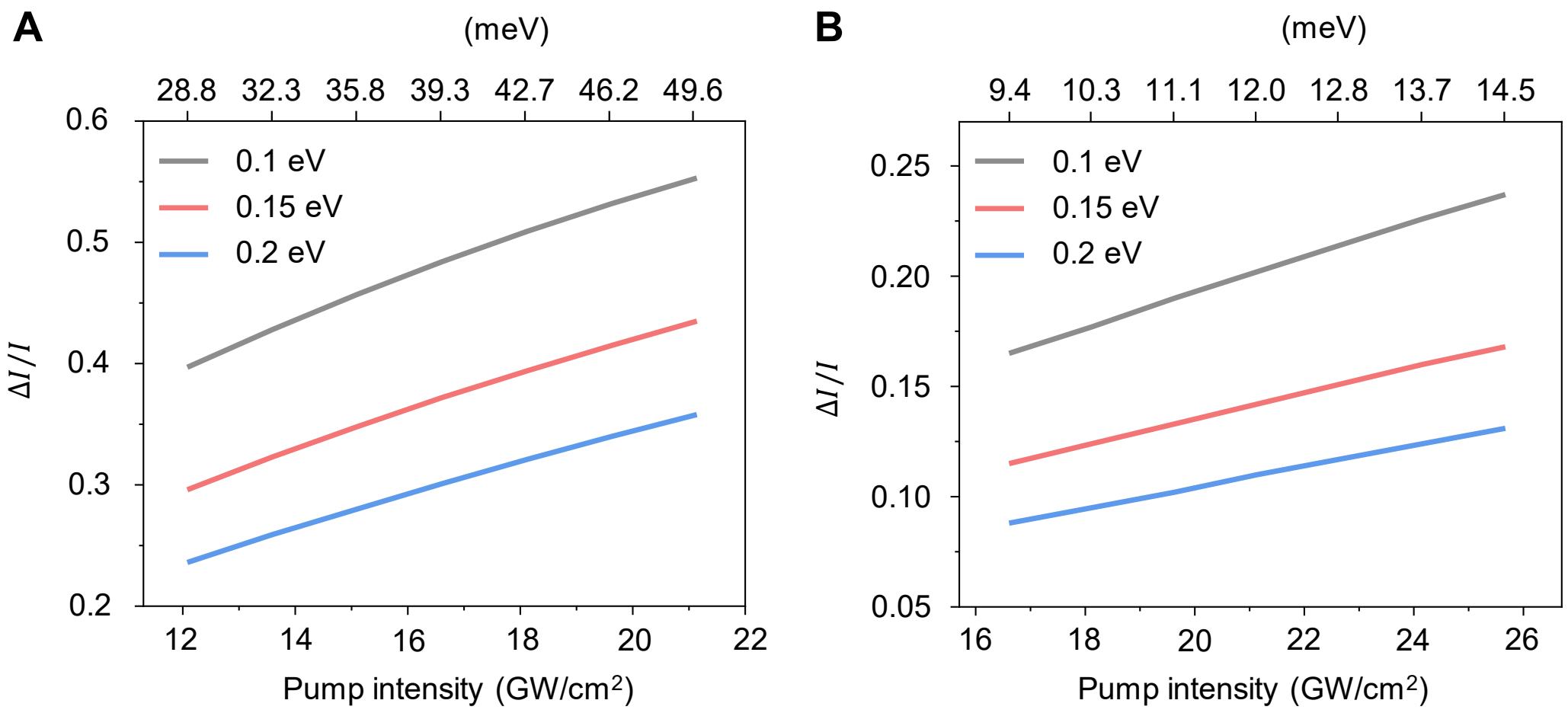

Fig. S2 Transient SHG's dependence on the excitonic states. Optical Stark effect **(A)** and Bloch-Siegert effect **(B)** induced transient SHG in reciprocal **k**-states with $E_f -$

$2\hbar$ ~ 0.1 eV, 0.15 eV, 0.2 eV and Γ_f~3 meV.

In the above calculations, we consider the continuum-of-states is modulated in the SHG process, i.e., the SHG photon energy (~3.1 eV) lies above the bandgap, within a continuum of electronic states. Within this energy range, multiple states may satisfy the parity selection rules required for SHG and be subject to modulation by the pump-induced energy shifts. Although the exact identities of these states remain uncertain, their collective response is well captured in our model as shown in Fig. S2. In above calculations (Fig. S2), the final state energy E_f was treated as a free parameter to match the experimental magnitude of the SHG response, while preserving the underlying physical trend.

According to Equation (S1), the intermediate state is also possibly modulated in the SHG process. We note that the fundamental photon energy (~1.55 eV) is close to the A-exciton resonance energy (~1.85 eV). This proximity suggests that the A-exciton may serve as a real intermediate state in the SHG process. In this picture, the pump-induced energy shift ΔE modifies the intermediate-state energy E_m in equation (S1), resulting in a modulation of the SHG signal. To justify the validity of the intermediate A-exciton mechanism, we performed extensive additional calculations across a broader spectral range. By varying the probe wavelength (729 nm, 775 nm, and 827 nm), we examined the SHG change at different detuning energies (~0.2 eV, 0.3 eV, and 0.4 eV with respect to the A-exciton). Figure S3 shows that the SHG change ($\Delta I/I_0$) consistently decreases under the chiral pump, and the modulation trend remains robust across this entire detuning range. This evidence demonstrates that the Floquet-induced renormalization of the A-exciton state exerts a long-range and consistent influence on the nonlinear susceptibility $\chi^{(2)}$, which is an intrinsic feature of the Floquet-dressed susceptibility throughout the band-nesting continuum.

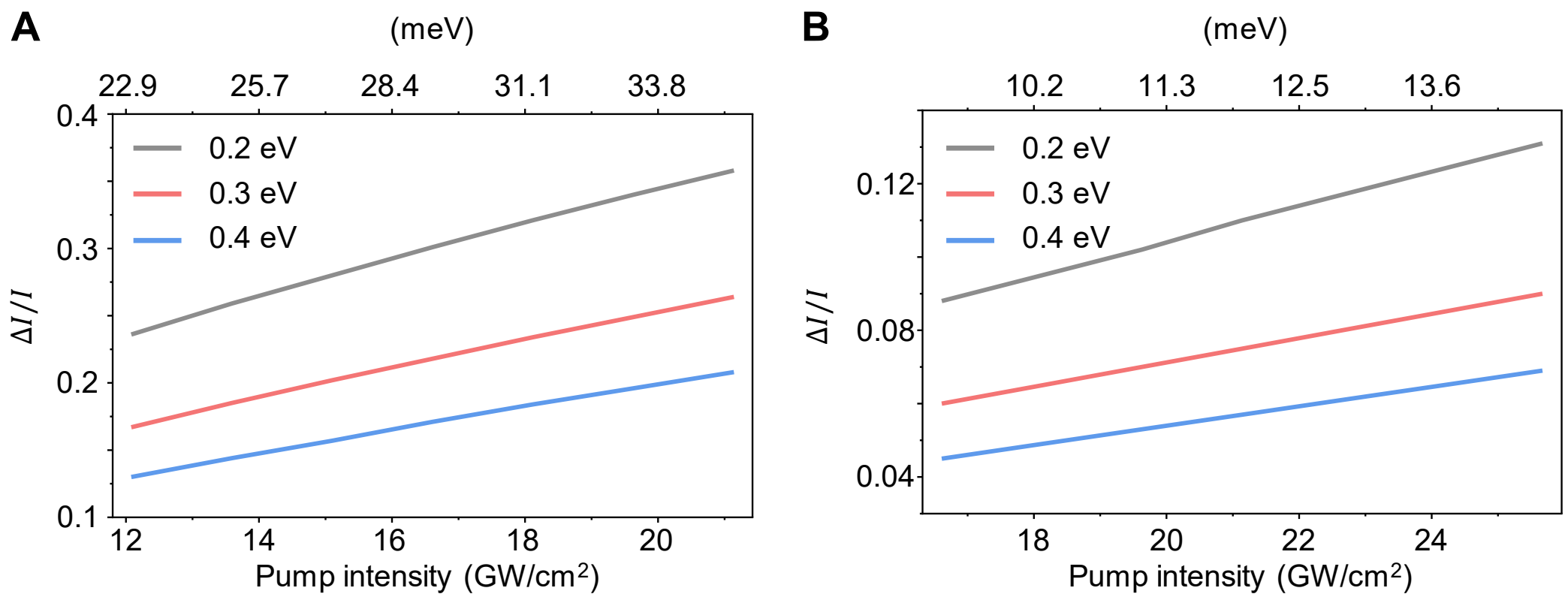

Fig. S3 Transient SHG's dependence on the excitonic states. Optical Stark effect **(A)** and Bloch-Siegert effect **(B)** induced transient SHG in K and K' valleys with E_m-$\hbar\omega$ ~0.2 eV, 0.3 eV, 0.4 eV and Γ_f ~3 meV.

Both scenarios yield results that are consistent with the experimental data,

supporting the robustness of our interpretation. According to the established length-gauge formalism for nonlinear optical responses in crystalline solids, the macroscopic second-order susceptibility $\chi^{(2)}$ tensor integrates distinct states across the entire Brillouin zone (BZ), $\chi^{(2)}_{\text{total}}= \chi^{(2)}_{\text{continuum}}+ \chi^{(2)}_{\text{Aexciton}}$ where different **k**-space regions cooperate via coherent optical interference. First-principles and empirical calculations specifically for monolayer MoS_2 have verified that different regions of the BZ dominate the $\chi^{(2)}$ spectrum at different photon energies. Although our probe photon energy of 3.1 eV is resonant with the diffuse continuum states and the C-exciton near the band-nesting region, the discrete A-exciton state possesses a large oscillator strength, and is governed by chiral optical selection rules ($\Delta m = \pm 1$). When the chiral (circularly polarized) Floquet pump field is applied, the interaction is governed by the symmetry of the driving field relative to the material's electronic wavefunction, distinguishing between co-rotating and counter-rotating driving field configurations. This near-resonant, chirality-dependent driving causes coherent quasi-energy modifications (i.e., optical Stark effect and Bloch-Siegert shift), which leads to a nonzero $\Delta\chi^{(2)}$. For example, the ~0.3 eV detuning from our 3.1 eV probe, the strong oscillator strength of A-exciton ensures its relative weight $\Delta\chi^{(2)}$ in the total $\chi^{(2)}$ tensor. As the SHG is a coherent process, the total intensity is the absolute square of the combined susceptibilities $I_0=| \chi^{(2)}_{\text{total}}|^2=| \chi^{(2)}_{\text{continuum}}+ (\chi^{(2)}_{\text{Aexciton}}+\Delta \chi^{(2)}_{\text{Aexciton}}|^2$. Expanding I_0 expression yields the cross-interference term that dictates the dynamic variation $\Delta I\sim 2\text{Re}[(\chi^{(2)}_{\text{continuum}})^*\Delta \chi^{(2)}_{\text{Aexciton}}]$. Therefore, we conclude that the chirality-selective SHG change is mainly modulated by intermediate A-exciton. Further experimental studies employing probe light with photon energies near the A-exciton resonance would be valuable for future investigations.

In the above simulations, we employed a damping rate of Γ_f = 3 meV to represent the homogeneous dephasing rate of coherent polarization during the ultrafast Floquet driving process. This choice allows for a high-resolution visualization of the coherent evolution of Floquet quasi-energy levels. However, it is noted that the excitonic linewidth observed in room-temperature experiments is typically 50 meV, primarily dominated by inhomogeneous broadening (e.g., phonon scattering, defects, and sample non-uniformity). To ensure that our theoretical conclusions are not sensitive to the specific choice of Γ_f, we performed sensitivity analysis by increasing the damping rate to Γ_f = 50 meV. As shown in Fig. S4, the calculated transient SHG change ($\Delta I/I_0$) with Γ_f = 50 meV exhibits a virtually identical trend to the results obtained with 3 meV. While the absolute modulation depth is increased due to the broader resonance, the fundamental chirality-selective features and the pump-intensity scaling laws remain robustly unchanged. This confirms that the damping rate affects only the quantitative magnitude rather than the qualitative physical mechanism of the chirality-selective Floquet engineering.

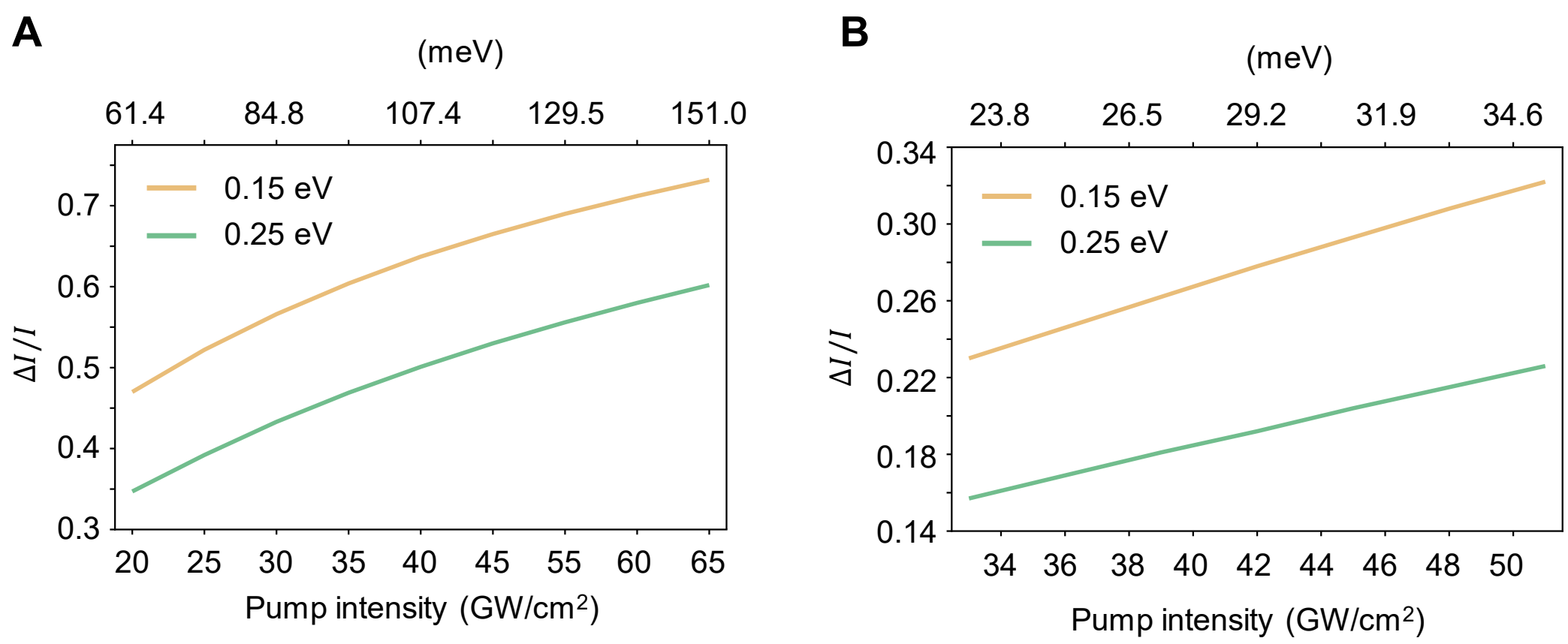

Fig. S4. Transient SHG's dependence on the excitonic states. Optical Stark effect **(A)** and Bloch-Siegert effect **(B)** induced transient SHG in reciprocal k-states with $E_f - 2\hbar\omega \sim 0.15$ eV, 0.25 eV and $\Gamma_f \sim 50$ meV.

S4. Chirality-selective modulation across pump wavelengths

To verify the robustness of the observed Floquet-induced SHG modulation, we compared the transient response driven by our primary pump (~1.24 eV, 1000 nm) to that by an alternative pump wavelength (~1.03 eV, 1200 nm), as shown in Fig. S5. The choice of pump photon energy is strategically constrained to the transparent regime of monolayer MoS_2 to avoid the real carrier population. Given that the A-exciton resonance is located at ~1.85 eV (Fig. S6D and S7B), both the 1000 nm and 1200 nm pumps provide a sufficiently large detuning to ensure a purely coherent light-matter interaction. As demonstrated in Fig. S5, the data obtained at ~1200 nm exhibits a high signal-to-noise ratio and a chirality dependence identical to those observed at ~1000 nm. Specifically, the characteristic contrast between σ^-/σ^- and σ^-/σ^+configurations remain robust. This consistency across different off-resonant pump energies confirms that the observed SHG modulation is an intrinsic consequence of chirality-selective Floquet engineering and is not tied to a specific parasitic resonance or material defect.

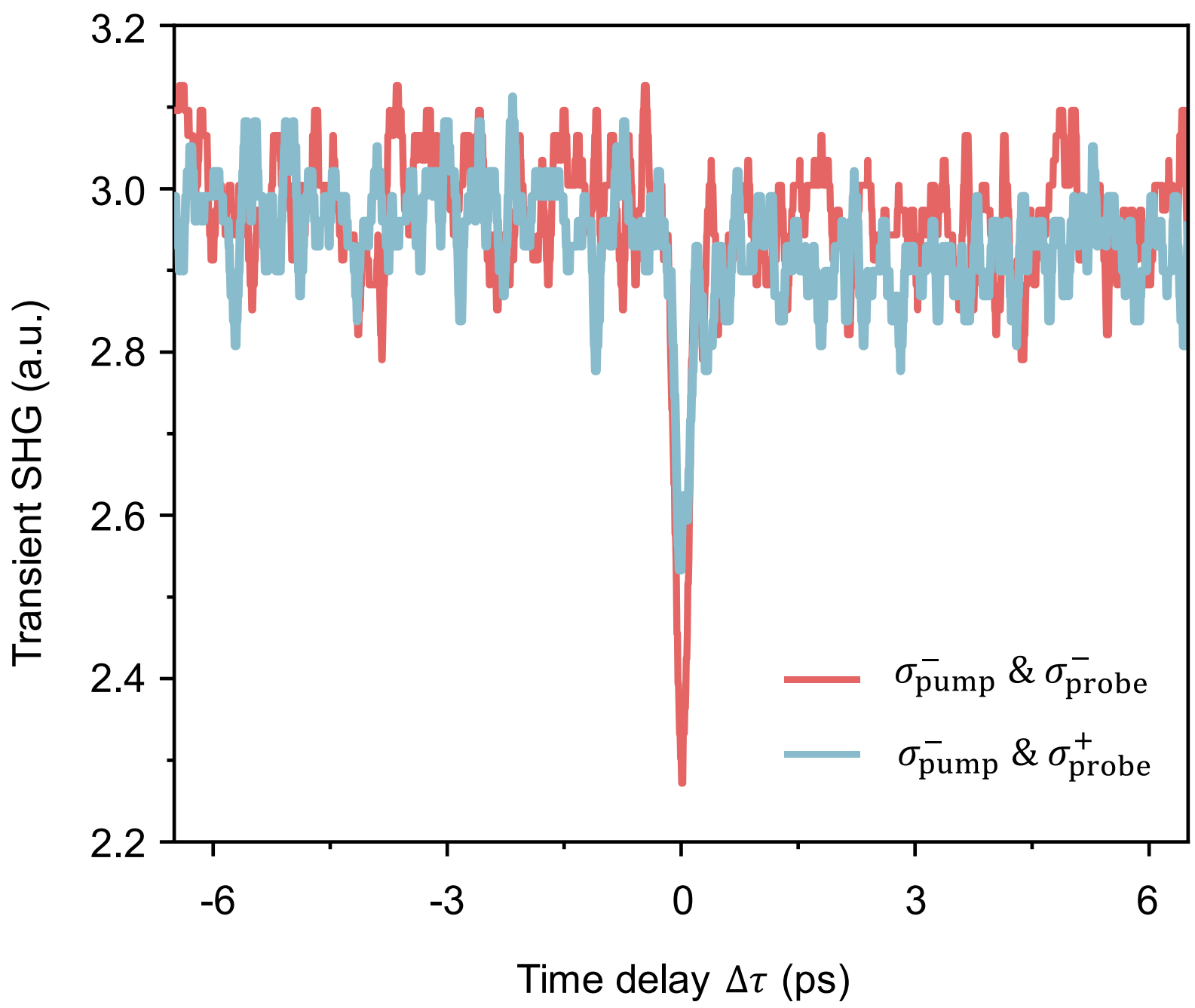

Fig. S5. Chirality-selective transient SHG. The transient SHG signals in CVD-grown monolayer MoS_2 are measured using a σ^- or σ^+ circularly polarized probe beam at a wavelength of ~800 nm in the presence of a σ^- circularly polarized pump beam at a wavelength of ~1200 nm. The intensities of the pump and probe beams are ~15 GW/cm^2.

S5 Chirality-selective SHG in mechanically exfoliated monolayer MoS_2

To verify that the observed chirality-selective Floquet response is an intrinsic property of monolayer MoS_2 rather than a defect-mediated effect, we conducted control experiments on high-quality mechanically exfoliated (ME) samples.
Figure S6A shows an optical microscope image of a monolayer MoS_2 flake prepared using the standard micromechanical cleavage and dry transfer technique. The monolayer region, outlined by the orange dashed line, was initially identified by optical contrast and further confirmed by spectroscopy characterization. Figure S6C shows the Raman spectra of ME monolayer, where the characteristic E^1_{2g} and A_{1g} peaks are observed at 386.1 cm^{-1} and 405.5 cm^{-1}, respectively. The frequency difference of 19.4 cm^{-1} is a clear signature of the monolayer thickness and high crystalline quality. The PL spectrum (Fig. S6D) exhibits sharp and prominent A-exciton (~667 nm) and B-exciton (~617 nm) resonance. The high photoluminescence (PL) intensity and narrow linewidth indicate superior optical quality with minimal non-radiative defect-induced quenching.

Transient SHG measurements on these high-quality ME samples (Fig. S6B) yield results that are remarkably consistent with those obtained from CVD-grown samples (Fig. 2B). This reproducibility of identical coherent and chirality-selective contrast demonstrates that the observed Floquet engineering is an intrinsic light-matter interaction dictated by the electronic band structure. The result effectively rules out structural defects, sulfur vacancies, or grain boundaries prevalent in CVD samples as the primary origin of the reported phenomena.

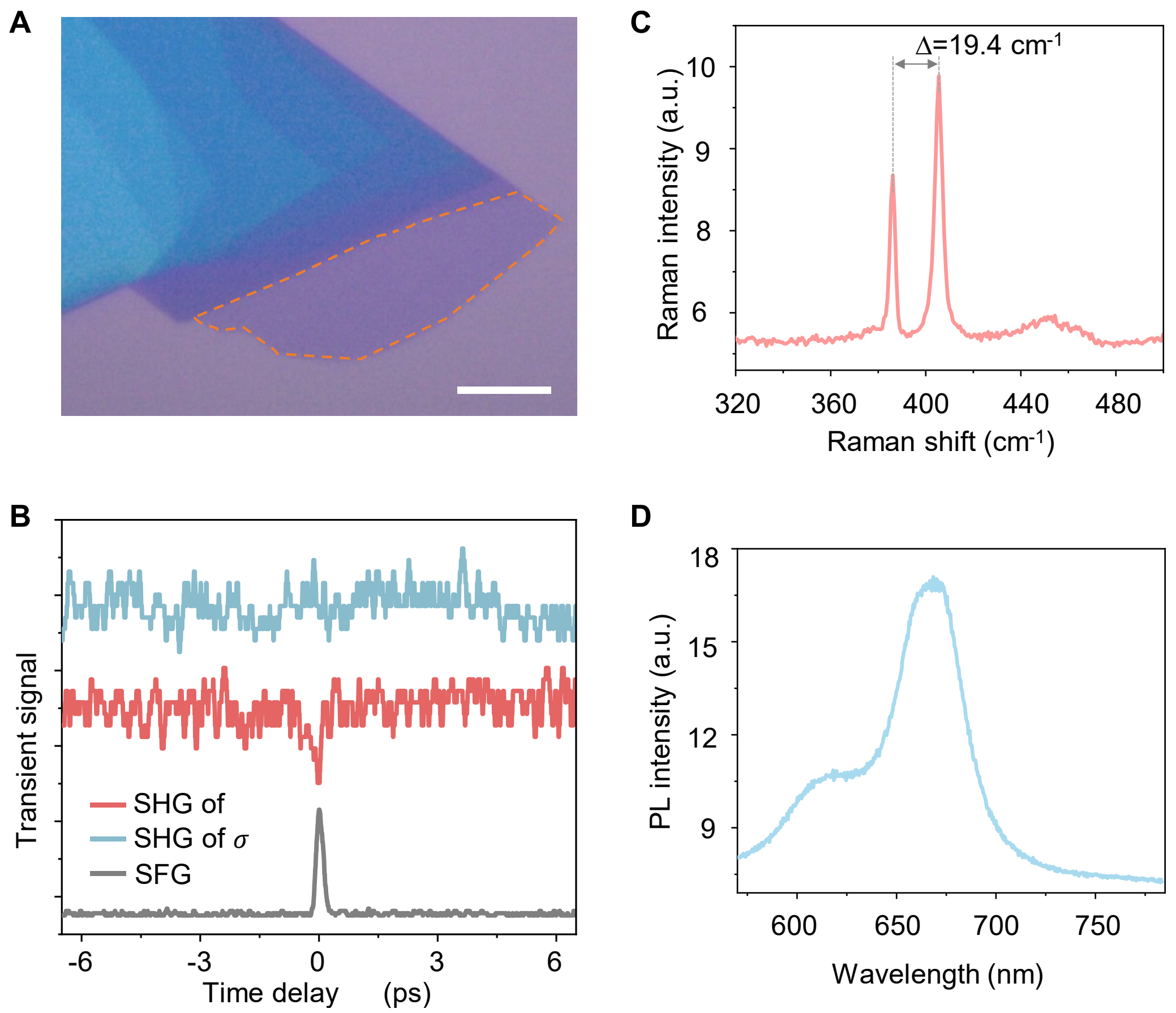

Fig. S6. Observation of Floquet-engineered chirality-selective transient SHG in mechanically exfoliated monolayer MoS_2. (**A**) A microscope image of mechanically exfoliated MoS_2, the orange dashed line enclosed area denotes the monolayer, the scale bar is 10 μm. (**B**) Transient SHG of the pump (blue line) and the probe (red line) beams from mechanically exfoliated monolayer MoS_2. Both beams are σ^- circularly polarized, and their SFG intensity (grey line) is recorded as a reference signal to determine the zero-time delay position between them. Note that the SHG and SFG signals are shifted vertically for a clear view. The intensities of the pump (at a wavelength of ~1200 nm) and probe beam (at a wavelength of ~800 nm) are ~5 GW/cm^2. (**C**) Raman shift of the mechanically exfoliated monolayer MoS_2 flake. (**D**) PL spectrum of the mechanically exfoliated monolayer MoS_2 flake.

S6 CVD grown MoS_2 characterization

Figure S7A gives the Raman spectrum of CVD grown monolayer MoS_2 with two characteristic peaks indicating the in-plane mode (E^1_{2g}) and out-of-plane mode (A_{1g}) at ~385.2 cm^{-1} and ~404.8 cm^{-1}. Figure S7B shows the measured PL spectrum of CVD grown monolayer MoS_2 with two prominent peaks corresponding to the A-exciton (at ~676 nm) and B-exciton (at ~626 nm), respectively.

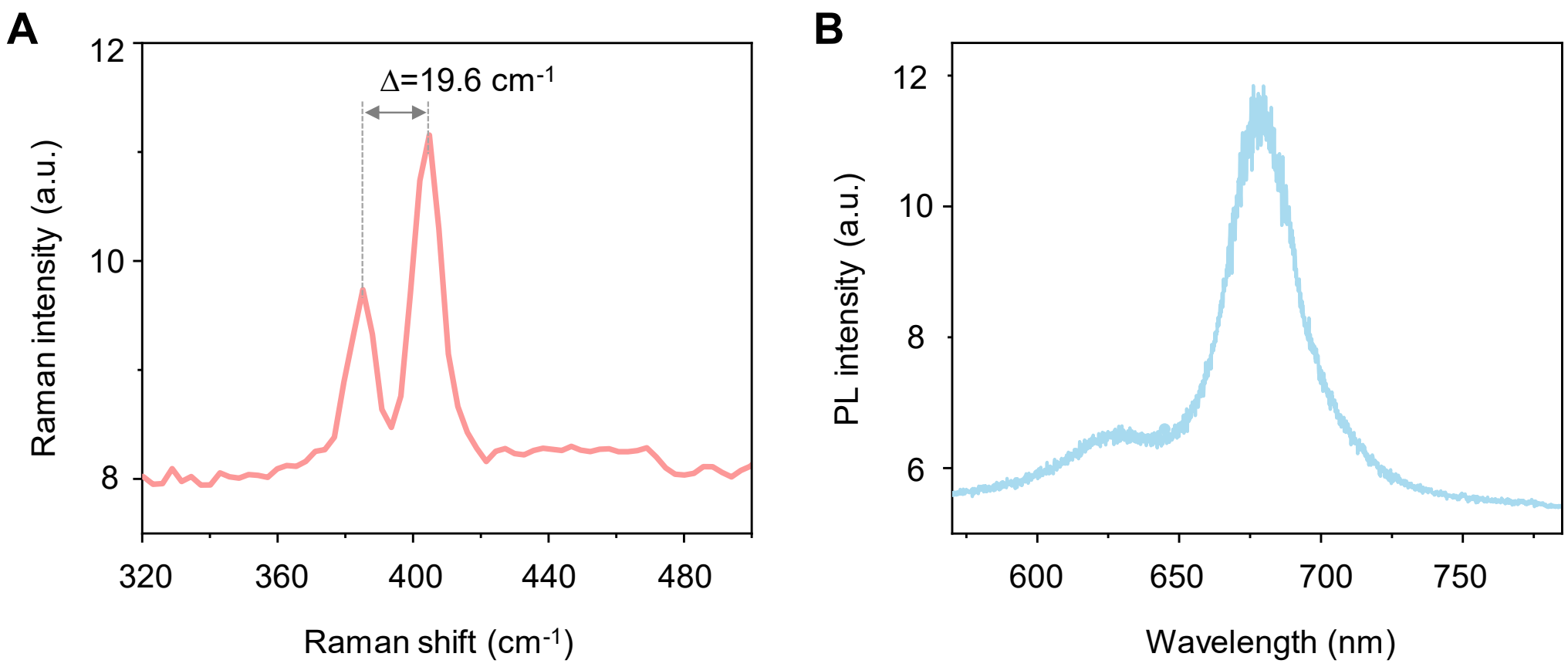

Fig. S7 CVD grown Monolayer MoS_2 characterization results. **(A)** Raman shift. **(B)** PL spectrum.
